# Analyzing Uncertainty of an Ankle Joint Model with Genetic Algorithm

**DOI:** 10.3390/ma13051175

**Published:** 2020-03-06

**Authors:** Adam Ciszkiewicz

**Affiliations:** Institute of Applied Mechanics, Cracow University of Technology, 31-155 Cracow, Poland; adam.ciszkiewicz@pk.edu.pl

**Keywords:** multibody system, statics, optimization, sensitivity, material and geometric parameters

## Abstract

Recent studies in biomechanical modeling suggest a paradigm shift, in which the parameters of biomechanical models would no longer treated as fixed values but as random variables with, often unknown, distributions. In turn, novel and efficient numerical methods will be required to handle such complicated modeling problems. The main aim of this study was to introduce and verify genetic algorithm for analyzing uncertainty in biomechanical modeling. The idea of the method was to encode two adversarial models within one decision variable vector. These structures would then be concurrently optimized with the objective being the maximization of the difference between their outputs. The approach, albeit expensive numerically, offered a general formulation of the uncertainty analysis, which did not constrain the search space. The second aim of the study was to apply the proposed procedure to analyze the uncertainty of an ankle joint model with 43 parameters and flexible links. The bounds on geometrical and material parameters of the model were set to 0.50 mm and 5.00% respectively. The results obtained from the analysis were unexpected. The two obtained adversarial structures were almost visually indistinguishable and differed up to 38.52% in their angular displacements.

## 1. Introduction

The ankle joint is a key structure of the lower body, which allows for the interaction of the lower limb with the ground [1]. Nevertheless, its acute injuries are among the most frequent ones of the musculoskeletal system [2]. The diagnosis and treatment of these injuries could be aided by biomechanical modeling of the joint. Furthermore, these models could also better the understanding of the ankle. However, one of the key issues to address to make the models clinically viable is the analysis of uncertainties caused by parameter determination and acquisition.

Typical multibody models of the ankle joint contain three types of elements: rigid bodies, which represent the bones; cable-like flexible or rigid links, which replace the ligaments; and contact pairs to substitute the cartilage between the bones. The parameters of these elements are usually based on either medical atlases or medical image scans—computer tomography (CT) or magnetic resonance (MR). Scans obtained from CT do not show the ligaments, therefore, the acquisition of the model parameters has to be subjectively performed by the researcher with the help of medical textbooks. Even if the soft tissue is visible in the image—as in MR—obtaining parameters involves, for instance, simplifying a spatially complex ligament attachment into a single point. This can be partially automated, but the process still has uncertainties. To make the models clinically-viable, it is important to understand how these uncertainties affect the results or—in other words—what outputs can be expected of the model, when the uncertainties in the parameter acquisition and determination are taken into account. Although, the trend to account for variability of the model parameters has already been noted in some recent studies on biomechanical modeling [3], most of the available studies on the ankle joint modeling [4,5,6,7,8,9,10,11,12,13] have not focused on this issue.

The most common approach to incorporate the sensitivity into biomechanical modeling is to vary the parameters of the model one at a time [3,14,15]. The studies, which considered combinations of multiple varying parameters, were much more rare. Depending on the system, other methods could be employed to study the influence of the model parameters on its outputs, these include: analytical or semi-analytical solutions [16,17], polynomial chaos expansion [18,19], a screening method [20], and a variety of different sampling methods [21,22,23].

A second option for accounting for uncertainties is to perform an uncertainty analysis, commonly used in electrical circuit design [24,25]. If the output of the model is a singular value, the result of the analysis is both the highest and the lowest value that the output can achieve under the assumed variability of the parameters. In this paper, the parameter sets, for which the models achieve these bounding values were referred to as the worst cases. Defining the worst-case for models, which output functions, for instance: a series of values over time, is more difficult. In circuit design, this issue is usually solved by finding a highest and a lowest value for each element of the output—namely, its lower and upper bounds or range, as in [25]. This analysis does not focus on the actual solution representing the worst case. It aggregates the worst cases, both high and low, in selected time steps, from multiple solutions. A different approach to this problem would be to actually find a parameter set, for which the model exhibits the most unusual behavior. This would provide more information on the behavior of the worst-case model during all the steps of its output and could be of importance in biomechanical models used for surgery and presurgical planning. Nevertheless, when the output is a highly nonlinear function, the definition of the worst-case behavior is not obvious. It could be the highest difference between the output of this worst-case and that of a reference, based on an assumed model or experimental results. However, the experimental results may not be available, while the output of the original model might be unreliable. Alternatively, the worst-case could be specified as ‘as close to zero as possible’ or ‘as high as possible’. Unfortunately, these objectives assume the shape of the output and can constrain the search. One other option is to omit references and specific objectives altogether and concurrently search for two models, which differ the most. This approach was proposed and tested for an ankle joint in this study.

It is worth mentioning that, regardless of the chosen approach, the limits on the parameter space have to be carefully selected to constrain the search space.

### Aim of the Study

The main aim of the study was to propose a method for analyzing uncertainty of an ankle joint model caused by parameter acquisition or determination. The main idea of the approach was to concurrently optimize two adversarial models in a way that maximizes the difference in their outputs. The optimization objective was formulated as a weighted L1-distance between the outputs of the two models. The search was performed with a variant of genetic algorithm.

The second aim of the study was to analyze the uncertainty of a planar model of the ankle proposed in [26]. This analysis was carried out with the bounds on geometrical and material parameters set to 0.50 mm and 5.00% respectively. The small bounds were selected to mimic the variability in both the subjective acquisition of geometrical parameters from medical images and the difficulties in obtaining material parameters for such models. To the best of my knowledge, such a method and an analysis have not been presented for biomechanical models of the ankle joint. The following sections contain a detailed description of approach and the obtained results.

## 2. Materials and Methods

### 2.1. Overview of the Proposed Approach

The main task of the procedure was to concurrently search for two adversarial structures, which differ the most in the vicinity of the parameter set of the original model. These structures represented the worst cases of the model due to parameter acquisition uncertainties. The search was posed an optimizational problem and solved with genetic algorithm. In this case, the decision variable vector contained two complete parameter sets—one for each structure. The objective function was computed as a distance between the outputs of the two adversarial structures.

To simplify the description of the method and to give it an actual context, the next subsection was focused on the ankle model, which was later analyzed with the approach.

### 2.2. Ankle Model Assumed to Verify the Procedure

**Figure 1:**
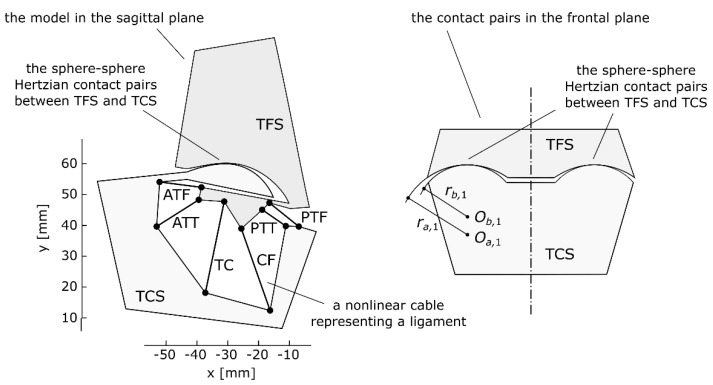
Assumed planar model of the ankle joint in the sagittal plane and a schematic representation of its spherical contact pairs, reproduced with permission from [26].

The planar ankle model employed in this study was based on [26]—see Figure 1. It contained two rigid bodies—one moving and one stationary. These bodies corresponded to the following bone segments: the talus/calcaneus for the moving body and the tibia/fibula for the basis. In total, six nonlinear cables connected the bodies. These cables substituted the following ligaments in the ankle: the anterior tibiotalar ligament (ATT), tibiocalcaneal ligament (TC), posterior tibiotalar ligament (PTT), anterior talofibular ligament (ATF), calcaneofibular ligament (CF), and posterior talofibular ligament (PTF). Furthermore, the bodies also interacted through Hertzian contact pairs of a sphere-sphere type, which represented the cartilage. The details concerning the mathematical equations for computing the forces of the elements and the equilibrium of such systems can be found in the following publications [26,27,28,29,30,31].

Internally, within the procedure, the models were solved for static equilibrium under the following external moment loads: *M_ext_* = −5.00: 5.00 Nm in 11 steps. The negative values of *M_ext_* corresponded to the motion clinically referred to as dorsiflexion, while the positive value represented plantarflexion.

### 2.3. Encoding the Adversarial Structures

**Figure 2:**
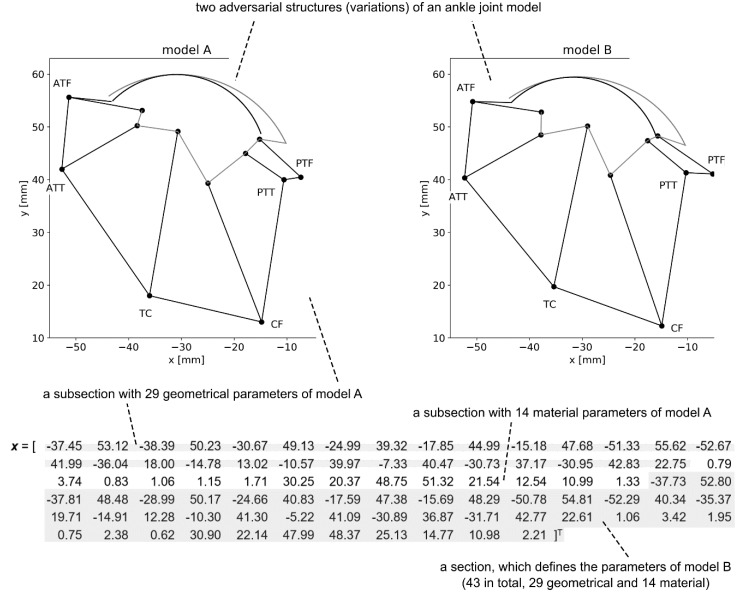
The proposed encoding of the adversarial structures—an example prepared based on an ankle joint model [26].

As mentioned previously, the approach was based on optimizing two models concurrently. In order to facilitate this, a custom decision variable vector was proposed. The vector consisted of two equal sections, each one containing a parameter set defining one of the structures. These sections could be further subdivided into geometrical parameters and material parameters subsections. In the case of the ankle model with 43 parameters assumed in this study the vector contained 86 (2 × 43) real-valued elements—see Figure 2. Out of the 43 parameters, 29 described the geometry, while 14 described the material behavior. To be more specific, the geometry section contained four coordinates of the attachments to the bone segments for each one of the ligaments, in total 24 (6 × 4), and additional five parameters describing the geometry of the contact pairs. The material section had two parameters per ligament, which described its behavior under the exponential model [32], and additional two parameters for the Young’s modulus and Poisson’s ratio of the contact pairs.

### 2.4. Objective Function

The main goal of the optimization procedure was to maximize the difference between the two adversarial structures encoded in the decision variable vector (see Section 2.3). Each computation of the objective was performed as follows. In the first step, the decision variable vector was split into two parameter sets of equal lengths. Then, two models—*A* and *B*—were created based on the sets and solved to obtain their output characteristics.

In order to formulate the objective function, some outputs of the model had to be selected. The approach did not constrain this selection—any output could be taken into account, including maximal strain in selected ligaments, linear stiffness of the model, and many others. In this study, the output was chosen to be the angular displacement over the external moment, as it is likely the most important attribute of a body joint. Based on this quantity, a difference between the two structures was obtained using a weighted L1-distance

(1) $$\sum_{i=1}^{m}w_{1i}\left|\Delta \theta_{A}^{}\left(M_{ext,i}\right)-\Delta \theta_{B}^{}\left(M_{ext,i}\right)\right|,$$

where *w_j_* is the weight *j* (here: *w*_1*i*_ = 1 for *i* = 1, …, (*m* − 1) and *w*_1*i*_ = 2 for *i* = [0, *m*]), Δ*θ_k_*(*M_ext,i_*) is the angular displacement of model *k* (*k* is either *A* or *B*) under the external moment load *M_ext,i_* (the considered loads were specified in the Section 2.2). In this case, the larger the value of Equation (1), the more different the structures were.

In order to transform the problem into a minimization, an additional minus sign was added before Equation (1). Based on that, the following objective function was formulated,

(2) $$\min_{x}h(x)=-\sum_{i=1}^{m}w_{1i}\left|\Delta \theta_{A}^{}\left(M_{ext,i}\right)-\Delta \theta_{B}^{}\left(M_{ext,i}\right)\right|+w_{2}not\_passed,$$

where *h* is the objective function, *w_j_* is the weight *j* (here: *w*_1*i*_ = 1 for *i* = 1, …, (*m* − 1) and *w*_1*i*_ = 2 for *i* = [0, *m*]), *w*_2_ = 10.00), Δ*θ_k_*(*M_ext,i_*) is the angular displacement of model *k* (*k* is either *A* or *B*) under the external moment load *M_ext,i_* (the considered loads were specified in the Section 2.2), *not_passed* is the number of loads for which the solver did not solve the models with desired accuracy (a sum for both of the adversarial structures; a similar approach was utilized in: [29,30,33]). The solutions, which contained models that were difficult to solve for all of the assumed loads were penalized with the second element of the objective function (2). The penalty was included with a relatively large weight in the objective function.

The models were solved with a least squares approach using Levenberg–Marquardt method from Scipy library [34]. The convergence settings for the solver were set to the default ones. However, an additional condition was imposed on the solver—the sum of the absolute values of the residual loads had to be less than 1.0 × 10^−10^ (see [26] for further details).

### 2.5. Optimization Procedure

A real-coded variant of genetic algorithm (RC-GA) [35] was used to minimize the objective function (2). This choice was not arbitrary as different variants of genetic algorithms [36], or evolutionary methods in general, have been proven effective in various optimizational problems [29,37,38,39,40,41]. Furthermore, typical local methods would not perform well in this problem—some of the structures did not converge, which made the objective function discontinuous and with many local minimums. RC-GA optimizes by checking multiple solutions in one iteration and then molding the best ones using the so-called genetic operators: selection, crossover, and mutation. This process is repeated multiple times until a convergence criterion is satisfied. In this study, the selection was performed with the roulette-wheel method [42]. The crossover was implemented as blx-alpha [43] (with alpha set to 0.5). Furthermore, non-uniform mutation [44] was applied to some solutions. The strength of the mutation decreased every iteration to improve convergence. The other settings for the algorithm were as follows: the number of generations set to 100, the population size set to 86, the elite fraction at 5%, the crossover fraction at 80% and 15% of the population obtained through mutation. More details regarding the algorithm can be found in [35].

### 2.6. Generating the Initial Population for the Algorithm

The initial population of the algorithm was generated randomly with the bounds based on the prior ankle model [26]. The lower and the upper bound for the geometrical parameters were obtained by adding and subtracting 0.50 mm respectively from the parameters based on [26]. The bounds for the material parameters were relative and set to ±5.00%, again based on [26]. These bounds were applied to both of the adversarial structures. Their values were selected to mimic the variability in both the subjective acquisition of geometrical parameters from medical images and the difficulties in obtaining material parameters for such models.

To improve the convergence of RC-GA one extra solution was injected into initial population. The solution contained two copies of the original model presented in [26]. As the solution had two same structures with the same outputs that solved for all the loading conditions, its objective value was, by definition, 0.00.

## 3. Results

This section was divided into two parts. The first part summarized the optimization process with the details regarding the optimization results during consequent generations as well as a general overview of the algorithm’s convergence. The second part was devoted to detailing the results of the sensitivity analysis performed on the ankle model.

### 3.1. Optimization Process

#### 3.1.1. Initial Runs of the Optimization

Optimization with genetic algorithms is a stochastic process and its results are dependent on the random number generator (RNG). This means that different results can be obtained for different seeds of the RNG. To obtain the best solution, the proposed procedure was run 10 times with different values of the RNG seed. Each run took on average 500 s using 10 threads of a Ryzen 5 1600 with 16 GB of RAM. In this section, the convergence of the run, which ended with the lowest value of the objective, was analyzed.

**Figure 3:**
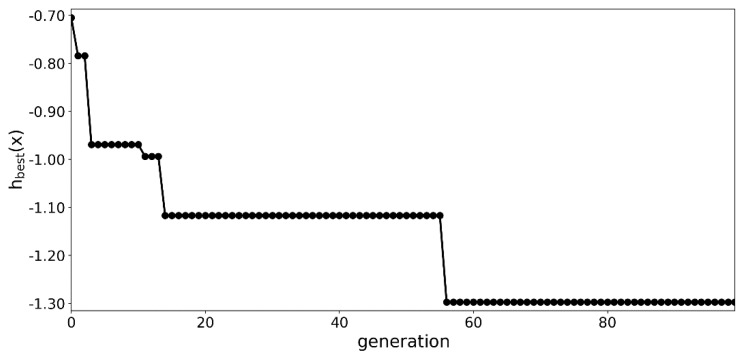
Value of the best fit specimen over the generations.

**Figure 4:**
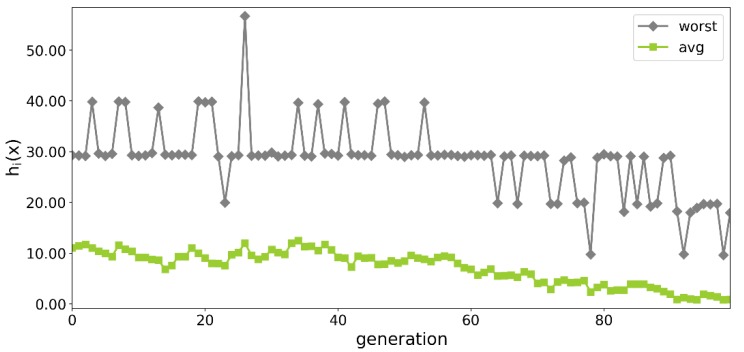
Value of the worst and the average specimen over the generations.

The optimization process was summarized in Figure 3 and Figure 4. A significant decrease in the value of the objective function was observed for in the first 15 generations as seen in Figure 3. In the following 85 generations, the algorithm continued to improve the best specimen, but at a much slower pace.

A steady convergence was also observed in the worst and the average solution. In the initial phase of the optimization, the structures from the worst solution did not carry between three to four of the assumed loading conditions.

In some of the later generations the worst solution contained structures, which did not transfer only one loading condition. A similar decreasing trend was observed for the average value of the objective function.

#### 3.1.2. An Extended Run with 2000 Generations

**Figure 5:**
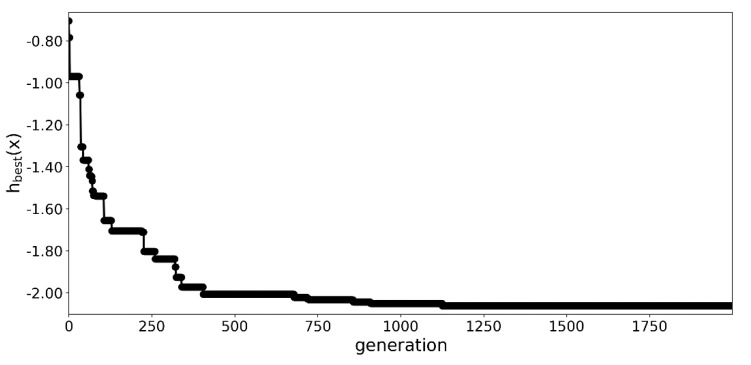
Value of the best fit specimen over the 2000 generations.

In the next part of the experiment, the optimization was rerun with the number of generations set to 2000—see Figure 5. Due to the significant numerical complexity, only the seed corresponding to the best run among 100 generations was employed for this simulation. In this case, the procedure needed almost 9200 s to complete. Even though the seed was the same as in the previous best run, a slight difference in optimization was observed in the initial 100 generations, due to the strength of mutation being dependent on the number of generations—see Figure 3 and Figure 5.

#### 3.1.3. Computing the Baseline

In order to assess the quality of the obtained results a baseline was necessary. In this study, this baseline was computed using a typical approach to sensitivity analysis found in many biomechanical studies [3]. The model was solved 43 times. During these runs, the parameters of the model were modified in a one-at-a-time fashion by either 0.5 mm or 5.00%, depending on the type of the parameter. This procedure was then repeated for modifiers of −0.5 mm or −5.00%. Additionally, during each run, the model with the modified parameter set was compared to the original model from [26] using the objective function used in optimization—see Equation (2). The best result obtained with this approach was presented in Table 1.

As seen in Table 1, the proposed optimizational procedure returned significantly better results. With regards to the common-sense solution—two copies of the original model with *h* of 0.0—see the Section 2.6, the optimizational approach was more than 5.5 times better at 100 generations and almost 9 times better at 2000 generations.

### 3.2. Analyzing the Uncertainty of the Ankle Model

**Figure 6:**
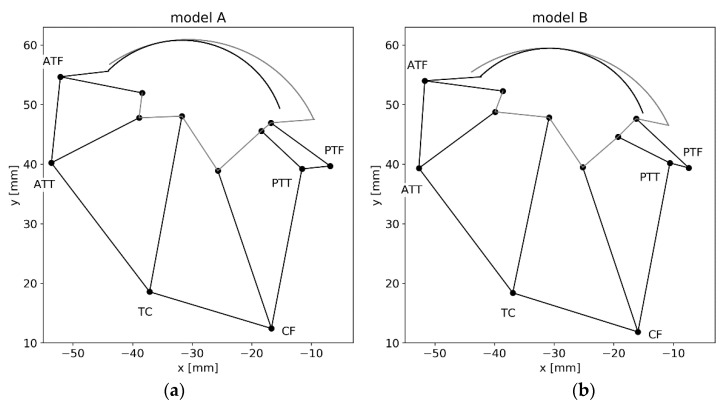
Two adversarial structures obtained with the algorithm for the ankle joint model (as seen in the prepared software). Only relevant parts of the contours of the bone segments were drawn. (**a**) Model A; (**b**) model B.

The two most differing adversarial solutions, obtained using the proposed procedure over 2000 generations, were presented in Figure 6. In terms of geometry, the difference between them was not significant. Such changes could be attributed to subjective preparation of the model geometry.

**Figure 7:**
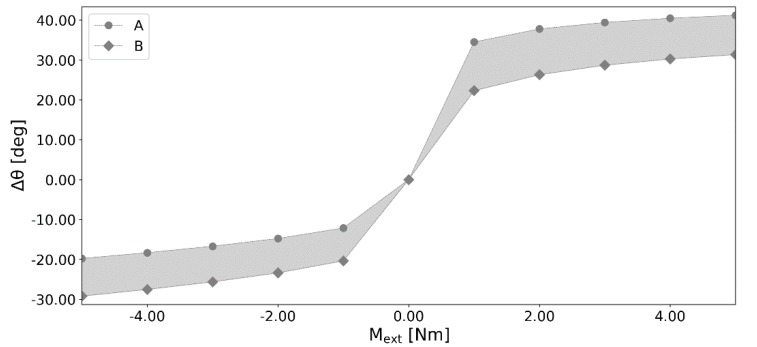
Angular displacement Δ*θ_i_* versus the external moment *M_ext_* for the two most different adversarial structures *A* and *B*.

Even though the differences in the geometry and the material parameters between the two obtained structures were relatively small, the resulting angular displacements were highly affected as seen in Figure 7. The relative difference between the structures was up to 27.18% (9.86 deg) in plantarflexion (positive moments) and 38.52% (9.44 deg) in dorsiflexion (negative moments)—see Table 2.

**Figure 8:**
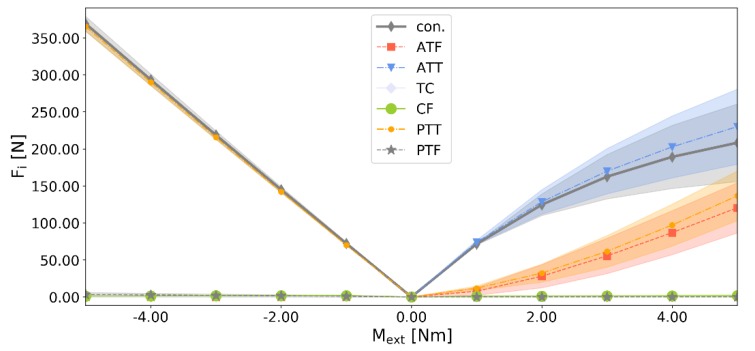
Values of the forces generated by the ligaments versus the external moment *M_ext_* for the two most different adversarial structures *A* and *B*, where: ‘con.’ is the magnitude of the contact force.

In terms of the loads generated by the ligaments (see Figure 8), significant differences between the two structures were observed in dorsiflexion. The highest absolute difference of 85.25 N (45.07%) was noted for the contact pairs, while ATT, ATF, and PTT experienced differences of 83.63 N (41.27%), 59.40 N (68.43%), and 56.75 N (58.43%) consecutively. The other ligaments remained mostly unaffected. The overall shapes of the curves remained similar to that of the base model from [26].

## 4. Discussion

### 4.1. Optimization Procedure

The obtained results showcased two important advantages of the proposed method. Firstly, when compared to approaches, which modify the model’s parameters one-a-at-time, the proposed method offered results up to 9 times better for extensive search. Even a relatively short run of 100 generations significantly outperformed the baseline approach. The second advantage of the proposed procedure was in its ability to account for multiple objectives in one search. To be more specific, a model of a healthy ankle joint displays two distinctive parts in its angular motion in the sagittal plane: the plantarflexion and the dorsiflexion. Both of these motions have to be accounted for in the objective. This makes it difficult to actually formulate the objective function in a way that does not constrain the search space. The proposed procedure addresses this problem with concurrent optimization of two models. In this case, the objective was simply formulated as the difference between the outputs of the two optimized models. No specific objectives, based on the two motions, were required to analyze the uncertainty of the model. Note that these specific objectives could also be optimized with the method, if there was a need for a more detailed analysis.

### 4.2. Effect of Uncertainties on the Ankle Model

An initial sensitivity analysis was carried out for the studied model of the ankle in [26]. In that paper, the first coordinate of TCS’s attachment of PTT was modified by 1.00 mm. This resulted in an angular displacement’s change of 3.67 deg and 1.40 deg in plantarflexion and dorsiflexion respectively. In contrast, the results obtained with the proposed method were far more striking. With parameter modifications bounded to only 0.50 mm (geometry) and 5.00% (material), the obtained difference within the angular displacements of the two adversarial structures was up to 27.18% (9.86 deg) in plantarflexion and 38.52% (9.44 deg) in dorsiflexion.

The obtained results clearly show how sensitive the model presented in [26] is to its geometrical and material parameters and their uncertainties. Additionally, it has to be emphasized that the allowed bounds on the geometry were only at 0.50 mm. Such a small difference could easily be attributed to subjective preference during parameter selection from medical images.

Future work should be focused on extending the analysis to feature different patient-specific parameter sets. With the current results, it is not clear if the model is only sensitive within the vicinity of the assumed parameter set [26] or if it is its inherent characteristic—observable for the majority of the patient-specific versions of the model.

## 5. Conclusions

In this study, a novel optimizational extension to the existing true-worst case analysis was proposed. The main idea of the approach was to concurrently optimize two adversarial structures. The objective was formulated as a weighted L1 distance between the output characteristics of the two structures and favored solutions in which the models differed greatly in terms of angular displacements. The problem was solved as a minimization with real-coded genetic algorithm.

The proposed procedure was used to analyze the effects of uncertainty of a planar model of the ankle with flexible links. The results of the analysis were striking. The two obtained structures, which differed only by ±0.50 mm and ±5.00% in geometrical and material parameters respectively, displayed vastly different angular displacements. While the overall ranges of motion for the structures were comparable, the differences were highly visible in the two major parts of the ankle’s motion—dorsiflexion and plantarflexion. The difference between the obtained structures was up to 38% in the angular displacement. The model was proven to be highly sensitive to small changes of its parameters. While these conclusions are directly applicable to the assumed model, it would be advisable to test different models of the ankle with similar structural elements—i.e., flexible links—under uncertainties.

On the other hand, the obtained results also proved the effectiveness of the proposed approach. The method is general and could be applied to different mechanical models, including finite element models. The future work could be devoted to testing the sensitivity of the method itself as well as performing a more complete analysis for the ankle model, which takes into account different starting parameter sets from patient-specific models.

## Figures and Tables

**Table 1:** Baseline obtained with one-at-a-time parameter modification compared to the results obtained with the proposed optimizational procedure.

	Baseline	Optimization after 100 gen.	Optimization after 2000 gen.
Value of the objective *h*(*x*) (obtained using Equation (2))	−0.23	−1.30	−2.06

**Table 2:** Maximal angular displacement Δ*θ_i_* in plantarflexion and dorsiflexion for the two most different adversarial structures *A* and *B*.

	Δ*θ_A_* (deg)	Δ*θ_B_* (deg)	*Avg*^1^ (deg)	*abs_diff*^1^ (deg)	*rel_diff*^1^ (%)
*M_ext_* = 5.00 Nm	41.21	31.35	36.28	9.86	27.18
*M_ext_* = −5.00 Nm	−19.79	−29.23	−24.51	9.44	38.52
range of motion	61.00	60.58	60.79	0.42	0.69

^1^*avg*—average; *abs_diff*—absolute difference, *rel_diff*—relative difference, computed as: (*abs_diff*/|*avg|)* × 100%.
